# Neuroimmune proteins can differentiate between tauopathies

**DOI:** 10.1186/s12974-022-02640-6

**Published:** 2022-11-19

**Authors:** Jonathan D. Cherry, Zach H. Baucom, Kaleb G. Eppich, Daniel Kirsch, Erin R. Dixon, Yorghos Tripodis, Kevin F. Bieniek, Kurt Farrell, Kristen Whitney, Madeline Uretsky, John F. Crary, Dennis Dickson, Ann C. McKee

**Affiliations:** 1 VA Boston Healthcare System, 150 S. Huntington Ave., Boston, MA 02130 USA; 2 Department of Pathology and Laboratory Medicine, Boston University School of Medicine, Boston, MA USA; 3 Boston University Alzheimer’s Disease and CTE Center, Boston University School of Medicine, Boston, MA USA; 4 Department of Biostatistics, Boston University School of Public Health, Boston, MA USA; 5 Department of Pathology, UT Health San Antonio, San Antonio, TX USA; 6 Gleen Biggs Institute for Alzheimer’s and Neurodegenerative Diseases, UT Health San Antonio, San Antonio, TX USA; 7 Department of Pathology, Icahn School of Medicine at Mount Sinai, New York, NY USA; 8 Department of Artificial Intelligence, Icahn School of Medicine at Mount Sinai, New York, NY USA; 9 Nash Family Department of Neuroscience, Icahn School of Medicine at Mount Sinai, New York, NY USA; 10 Friedman Brain Institute, Icahn School of Medicine at Mount Sinai, New York, NY USA; 11 Neuropathology Brain Bank and Research CoRE, Icahn School of Medicine at Mount Sinai, New York, NY USA; 12 Ronald M. Loeb Center for Alzheimer’s Disease, Icahn School of Medicine at Mount Sinai, New York, NY USA; 13 Department of Neuroscience, Mayo Clinic, Jacksonville, FL USA; 14 Department of Neurology, Boston University School of Medicine, Boston, MA USA

**Keywords:** Tau, Neuroinflammation, Tauopathies, Biomarkers, Immune, Neurodegeneration, CSF

## Abstract

**Background:**

Tauopathies are a group of neurodegenerative diseases where there is pathologic accumulation of hyperphosphorylated tau protein (ptau). The most common tauopathy is Alzheimer’s disease (AD), but chronic traumatic encephalopathy (CTE), progressive supranuclear palsy (PSP), corticobasal degeneration (CBD), and argyrophilic grain disease (AGD) are significant health risks as well. Currently, it is unclear what specific molecular factors might drive each distinct disease and represent therapeutic targets. Additionally, there is a lack of biomarkers that can differentiate each disease in life. Recent work has suggested that neuroinflammatory changes might be specific among distinct diseases and offers a novel resource for mechanistic targets and biomarker candidates.

**Methods:**

To better examine each tauopathy, a 71 immune-related protein multiplex ELISA panel was utilized to analyze anterior cingulate grey matter from 127 individuals neuropathologically diagnosed with AD, CTE, PSP, CBD, and AGD. A partial least square regression analysis was carried out to perform unbiased clustering and identify proteins that are distinctly correlated with each tauopathy correcting for age and gender. Receiver operator characteristic and binary logistic regression analyses were then used to examine the ability of each candidate protein to distinguish diseases. Validation in postmortem cerebrospinal fluid (CSF) from 15 AD and 14 CTE cases was performed to determine if candidate proteins could act as possible novel biomarkers.

**Results:**

Five clusters of immune proteins were identified and compared to each tauopathy to determine if clusters were specific to distinct disease. Each cluster was found to correlate with either CTE, AD, PSP, CBD, or AGD. When examining which proteins were the strongest driver of each cluster, it was observed the most distinctive protein for CTE was CCL21, AD was FLT3L, and PSP was IL13. Individual proteins that were specific to CBD and AGD were not observed. CCL21 was observed to be elevated in CTE CSF compared to AD cases (*p* = 0.02), further validating the use as possible biomarkers. Sub-analyses for male only cases confirmed the results were not skewed by gender differences.

**Conclusions:**

Overall, these results highlight that different neuroinflammatory responses might underlie unique mechanisms in related neurodegenerative pathologies. Additionally, the use of distinct neuroinflammatory signatures could help differentiate between tauopathies and act as novel biomarker candidate to increase specificity for in-life diagnoses.

**Supplementary Information:**

The online version contains supplementary material available at 10.1186/s12974-022-02640-6.

## Background

One of the most prevalent types of neurodegenerative pathologies are tauopathies, a class of neurodegenerative disease that demonstrate pathologic accumulation of hyperphosphorylated tau (ptau) in the brain [1]. The most common tauopathy is Alzheimer’s disease (AD), but diseases like chronic traumatic encephalopathy (CTE), progressive supranuclear palsy (PSP), corticobasal degeneration (CBD), and argyrophilic grain disease (AGD) are significant health risks as well. Neuropathologically, ptau can be observed to have a unique regional progression and cellular aggregation patterns specific to each disease [2–9]. However, the mechanisms behind neuropathology are still unknown and it is unclear if there are early shared mechanisms common among diseases. Additionally, tauopathies can only be diagnosed after death and lack specific diagnostic biomarkers. While the use of ptau in fluids, like blood and cerebrospinal fluid (CSF), has shown to be a possible marker of general neurodegenerative processes, additional biomarkers are still needed that can sufficiently differentiate between tauopathies and aid antemortem diagnosis [10–12].

As there is greater understanding of neurodegenerative disease processes, it has become increasing clear that neuroinflammatory changes might be specific among diseases. The idea that the immune response is either inflammatory or anti-inflammatory has been proven to be far too simplistic [13, 14]. Recent single cell sequencing studies have challenged such dichotomous classification and suggested that the immune response present during neurodegenerative diseases is incredibly complex and diverse depending on the disease [15–18]. Furthermore, additional work has demonstrated that microglial mediated inflammatory response appears to be tailored towards the specific insult [14]. These complex changes potentially offer the ability to find a “neuroinflammatory signature” and might prove to be a useful source of mechanistic targets important to disease pathogenesis and novel biomarker candidates. To that end, previous research has suggested that inflammatory related markers like CCL11 could distinguish between AD and CTE [19]. However, CCL11 is also increased with aging making it challenging to use in cohorts populated with primary aged individuals [19, 20]. Therefore, a deeper analysis of multiple diverse inflammatory or neuroimmune mediators might offer a powerful approach to identify novel proteins that could discriminate between tauopathies.

Utilizing grey matter from the anterior cingulate cortex, a 71 immune-related protein multiplex ELISA was performed comparing the tauopathies AD, CTE, PSP, CBD, and AGD to identify novel neurodegenerative pathways and biomarkers. To narrow down the candidate biomarker list, an unbiased partial least square regression model was used to identify clusters of specific proteins that best correlated to each disease group while controlling for age at death and gender. Using the top distinct proteins, receiver operator curves and binary logistic regression analyses were performed to demonstrate the sensitivity, specificity, and predictive power for each protein. Finally, to validate the ELISA results and show efficacy on their use as novel candidate biomarkers, the top distinctive protein for CTE was used to examine the ability to identify disease in CSF compared to AD. Overall, due to the inherent complex nature of the neuroinflammatory response, this work suggests that in-depth analyses of neuroinflammatory components can reveal (1) novel mechanisms of disease specific to each tauopathy and (2) identify better fluid biomarkers to more accurately identify and discriminate tauopathies in life.

## Materials and methods

### Subjects

Postmortem fresh frozen human brain tissue was obtained and processed from 127 individuals as previously described [19]. Additionally, 29 samples of cerebrospinal fluid (CSF) were obtained as previously described [11]. Cases were evaluated from the BU CTE Center, BU Alzheimer’s Disease Center, Framingham Heart Study, and Mayo Clinic Brain Banks. Next of kin provided written consent for participation and brain donation. IRB approval was obtained through the Boston University Alzheimer’s Disease and CTE Center and Mayo Clinic brain bank. Cases were assessed for neurodegenerative diseases using well established criteria for AD, neocortical Lewy body disease, frontal temporal lobar degeneration, motor neuron disease, CTE, PSP, CBD, and AGD [2–8]. For AD, cases were included if they had a Braak Stage of at least 3 and a CERAD score of at least 2. Cases were divided into 5 groups based on the presence of either AD, CTE, PSP, CBD, or AGD. Each group did not have comorbid neurodegenerative diseases or overlapping pathologic hallmarks. For brain tissue homogenate, 40 cases had a diagnosis of CTE, 28 had AD, 20 had PSP, 20 had CBD, and 19 had AGD. For CSF, 14 cases had CTE and 15 had AD. Case selection represented a convenience sample set from each respective brain banks to best age and gender match between the 5 groups used in the study. However, as no females have been diagnosed with CTE, only males were selected. A full breakdown of sample demographics for brain homogenate samples is in Table 1 and for CSF samples in Table 2.

**Table 1:** Demographics for brain homogenate samples

	CTE	AD	PSP	CBD	AGD
*n*	40	28	20	20	19
Age at death (years)	70.3 ± 11.3	79.7 ± 9.4	71.2 ± 4.4	64.8 ± 5.3	80.5 ± 6
Gender (male/female)	40/0	13/15	10/10	11/9	11/8
CERAD score	0.1 ± 0.3	2.9 ± 0.4	0 ± 0	0 ± 0	0 ± 0

Values represented as mean ± SEM

**Table 2:** Demographics for CSF samples

	CTE	AD
*n*	14	15
Age at death (years)	72.5 ± 15.5	83.5 ± 8.5
Gender (male/female)	14/0	5/10
CERAD score	0.2 ± 0.4	2.5 ± 0.5

Values represented as mean ± SEM

### ELISA

Frozen tissue from the anterior cingulate cortex was harvested using methods previously described [21]. Anterior cingulate cortex was selected as it is a region that can be affected by ptau in all 5 of the diseases [22–26]. Isolated frozen grey matter was homogenized using the Precellys Evolution + Cryolys Evolution system (Bertin Bioreagent) as per manufacturer’s instructions. Briefly, 500 mg of brain tissue was placed into a 7 mL CK28 Precellys lysis tube containing 5 mL of RIPA buffer. The tubes were placed into the Precellys Evolution + Cryolys Evolution and run at 6500 rpm 3 × 20 s with a 15 s break at 4 ℃. Samples were then centrifuged at 3000×*g* at 4 ℃ for 10 min. The supernatant was removed and stored. Samples were then diluted with RIPA buffer to the final concentration of 4 mg/mL. Diluted samples were sent to Eve Technologies and run on the Human Cytokine/Chemokine 71-plex discovery assay array (HD71). 8 protein targets were not expressed in any of the samples and excluded from the analyses.

To validate candidate biomarkers in a more relevant setting, postmortem CSF was obtained from 29 cases and prepared as previously described [11]. Samples were run on the CCL21/6CKine DuoSet ELISA kit from R&D systems as per manufacturer’s instructions. Plates were imaged using a SpretraMax M3 imager (Molecular Devices).

### Statistics

To identify disease specific proteins using an unbiased approach, Partial Least Square (PLS) regression analysis was carried out with a Kendall Correlation. The aim of PLS is to measure the inter-relatedness between two blocks of variables. For this analysis, one block is made up of the 5 contrasts of interest (CTE vs all other neuropathologies, AD vs all other neuropathologies, etc.). The other block contains the ELISA protein levels after adjusting for age and gender. To carry out the PLS, singular value decomposition (SVD) is conducted on a partial correlation matrix between each contrast and ELISA proteins after adjusting for age and gender, where each column corresponds to a contrast and each row corresponds to a biomarker. The resulting matrices from SVD provide insight into how contrasts and ELISA proteins load to underlying constructs. Each protein was given a correlation number that represented how much a specific protein contributes to each neuropathologic disease. The advantage of using the PLS regression is that it reduces the number of target proteins for analysis, so a multiple comparison correction across all 71 proteins in the panel was not needed. To better understand if gender influenced the results, a sub-analysis using just males was also performed. Receiver operating characteristic (ROC) curve and binary logistic regression analyses were used to determine the specificity and sensitivity of the top candidate proteins identified from PLS analysis. For ROC analysis, each disease was compared against all other pathologies (i.e. CTE cases vs all other diseases). ROC was analyzed for each protein individually. Multiple comparison correction was applied to the ROC results. Binary logistic regression was used to determine if proteins were predictive of distinct tauopathies when accounting for age and gender differences. Finally, Mann–Whitney tests were used to compare between AD and CTE CSF. Descriptive statistics, ROC curve, and Mann–Whitney tests were generated using SPSS (v26, IBM).

## Results

### Partial least square regression model identified 5 clusters of proteins that correlated with distinct tauopathy

**Fig. 1:**
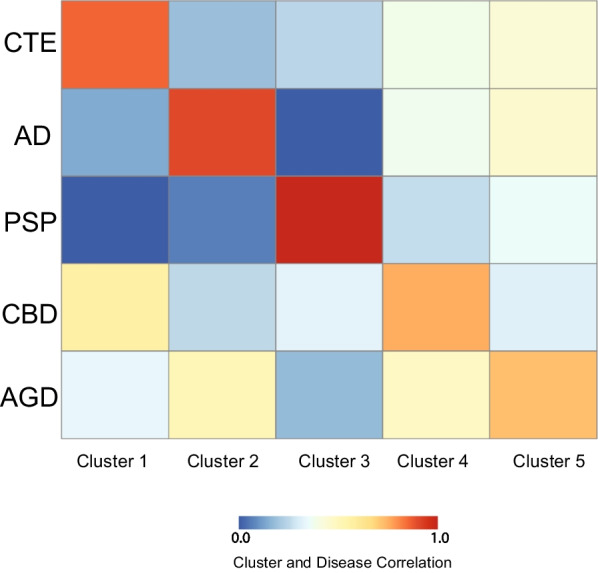
Partial Least Square regression identifies clusters of proteins that correlate with each tauopathy. Using the partial least square regression analysis, five clusters of ELISA proteins were identified and compared against each tauopathy to determine degree of correlation. Each comparison was a contrast of a single tauopathy against all other neuropathologies. Blue represents low correlation and red represents high correlation. Comparisons are adjusted for age at death and gender. Cluster 1 was most correlated with CTE, Cluster 2 with AD, Cluster 3 with PSP, Cluster 4 with CBD, and Cluster 5 with AGD

To identify possible biomarkers or mechanisms that are distinct among CTE, AD, PSP, CBD, and AGD, a 71 immune related protein multiplex ELISA was performed on anterior cingulate cortex grey matter. Using PLS regression analysis, 5 clusters of proteins were identified and correlated against each tauopathy to determine what clusters were related to specific disease (Fig. 1). Age and gender were included into the model to correct for differences. However, it is important to note that no females were present in the CTE group. Cluster 1 was most correlated with CTE, Cluster 2 was most correlated with AD, Cluster 3 was most correlated with PSP, Cluster 4 was most correlated with CBD, and Cluster 5 was most correlated with AGD. Although clusters 4 and 5 demonstrated higher specificity for CBD and AGD respectively, there was minor overlap with the other tauopathies as well.

**Fig. 2:**
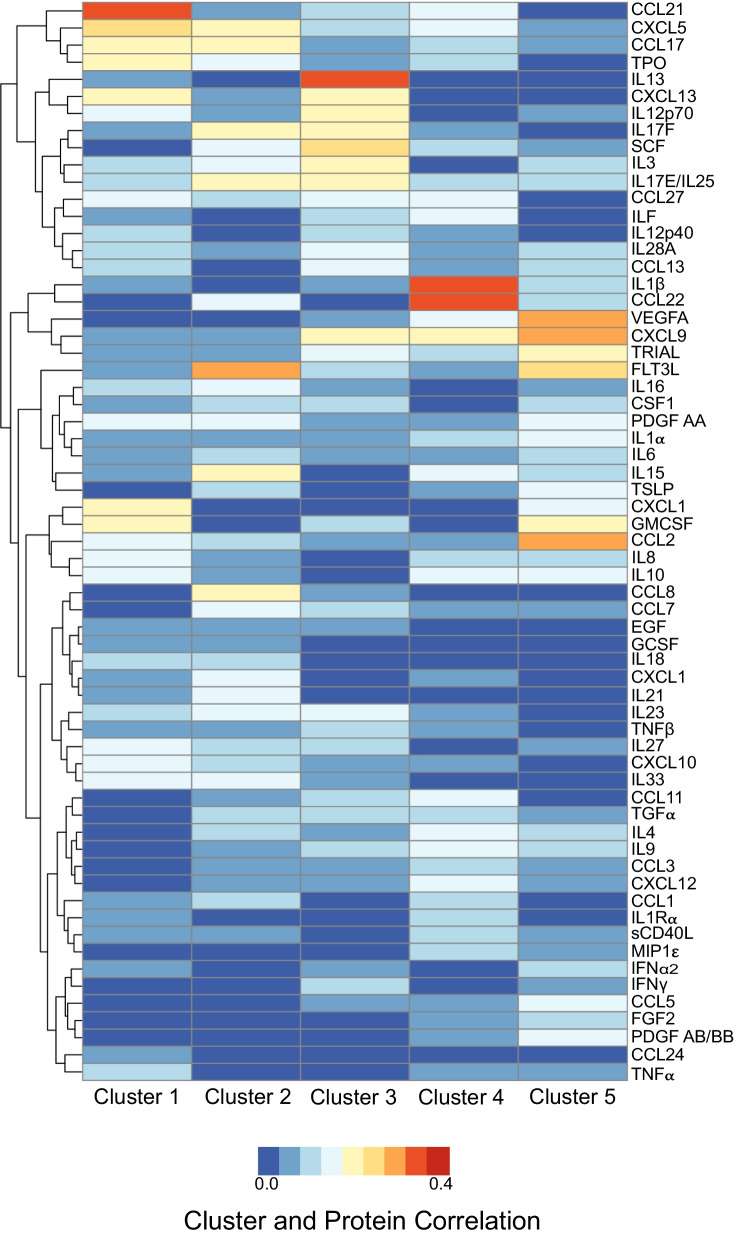
Correlation of each cluster to ELISA proteins identifies which immune factors are most specific to each disease. Singular value decomposition (SVD) was conducted on a partial correlation matrix between each cluster and ELISA proteins after adjusting for age and gender, where each column corresponds to a cluster and each row corresponds to an ELISA protein. The resulting heatmap demonstrates which immune proteins best correlate to each cluster/disease. From this heatmap, the top 5 candidate proteins that are distinct for each cluster/disease were selected. Blue represents low correlation and red represents high correlation

Next, each cluster was analyzed to examine which specific proteins were driving the cluster classification (Fig. 2) and the top 5 proteins for each cluster were identified. The full list of proteins is presented in Additional file 2: Table S1. The most correlated protein for Cluster 1/CTE was CCL21 (0.36), then followed by CXCL5 (0.27), CXCL13 (0.24), GMCSF (0.25), then CCL17 (0.23). In Cluster 2/AD, the highest correlated protein was FLT3L (0.31), then followed by IL17F (0.25), CCL17 (0.23), IL15 (0.23), then IL17E/IL25 (0.21). In Cluster 3/PSP, the highest correlated protein was IL13 (0.37), followed by SCF (0.25), CXCL13 (0.25), CXCL9 (0.24), then IL3 (0.24). In Cluster 4/CBD, the highest correlated protein was IL1β (0.37), followed by MDC (0.37), CXCL9 (0.21), ILF (0.2), then VEGFA (0.19). Finally, in Cluster 5/AGD the highest correlated protein was VEGFA (0.33), followed by CCL2 (0.32), CXCL9 (0.31), FLT3L (0.28), then GMCSF (0.22).

Although gender was controlled for as covariate in the PLS regression, a sub-analysis of just males was performed. The full list and order of proteins for the sub-analysis is present in Additional file 3: Table S2. The top proteins for Cluster 1/CTE remained consistent with CCL21 (0.31) as the highest associated protein. Cluster 3/PSP also was consistent with IL13 as the highest associated protein. However, there was some differences across the other 3 groups. In Cluster 2/AD the highest male associated protein was IL23 (0.27), then CCL2 (0.24), followed by FLT3L (0.24). CXCL9 (0.35) was the highest associated protein in both Cluster 4 and 5. Overall, given that the sample size was severely reduced when excluding females, the findings in the male only subset was fairly consistent with the mix gender full analysis.

### The top 5 proteins in each cluster could identify distinct neurodegenerative pathologies

**Fig. 3:**
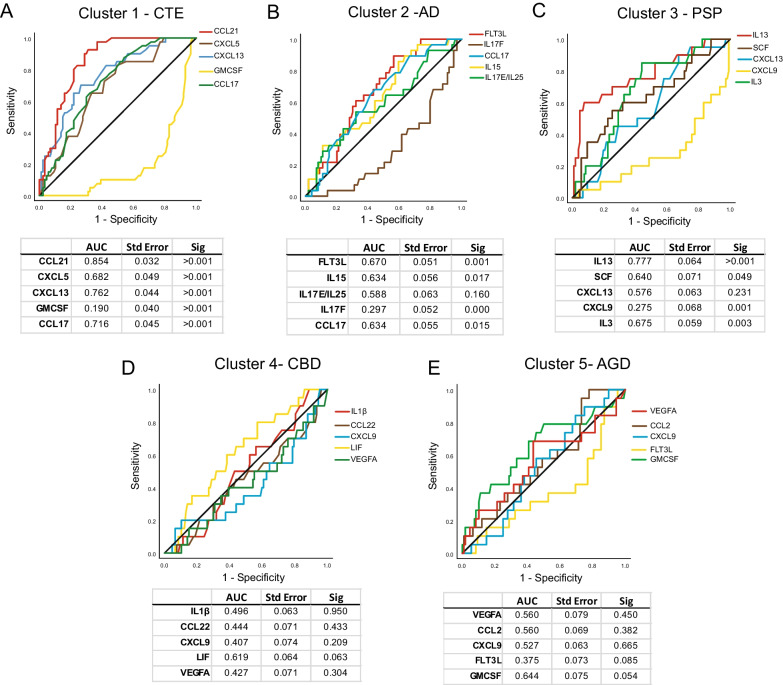
Receiver operator characteristics (ROC) curve demonstrates the top 5 candidate proteins for each cluster are specific and sensitive to identify tauopathies. Using the PLS results and top 5 candidate proteins for each cluster, ROC curve was performed for A) CTE, B) AD, C) PSP, D) CBD, and E) AGD. The area under the curve (AUC), standard error, and significance is displayed below each graph. AUC values over 0.5 suggest positive association with each disease, while values under 0.5 represent negative association. The black line is the reference line. Multiple comparison correction set the significance threshold to *p* < 0.01

Using the top 5 proteins from each cluster in the full mixed gender analysis group, receiver operating curve (ROC) analyses was performed to examine how specific and sensitive each protein was for identifying distinct tauopathies (Fig. 3). Multiple comparison correction was performed and *p* < 0.01 was the new significance threshold. ROC analysis of the top 5 Cluster 1 proteins for the ability to identify CTE demonstrated that CCL21 had the highest area under the curve (AUC) (0.854, *p* < 0.001). CXCL5, CXCL13, GMCSF, and CCL17 all were significant as well (Fig. 3A). GMCSF was the only Cluster 1 protein that had a significant AUC under 0.5 demonstrating it is found at lower levels in CTE compared to the other diseases. The top 5 proteins in Cluster 2 were used to identify AD. FLT3L had the highest AUC (0.670, *p* = 0.001), followed CCL17 (0.634, *p* = 0.015). IL15 did not meet multiple comparison corrected significance (0.634, *p* = 0.017). IL17F (0.297, *p* < 0.001) had a significant AUC under 0.5 demonstrating decreased expression in AD compared to the other diseases (Fig. 3B). Next, the top 5 proteins in Cluster 3 were used to identify PSP. IL13 was observed to have the highest significant AUC (0.777, *p* < 0.001), followed by SCF (0.640, *p* = 0.49), and IL3 (0.675, *p* = 0.003). CXCL9 (0.275, 0.001) had an AUC under 0.5 demonstrating less protein expression in PSP compared to other diseases (Fig. 3C). Cluster 4 and Cluster 5 did not have any significant AUC values when used to identify CBD (Fig. 3D) and AGD (Fig. 3E), respectively.

The protein with the highest AUC values in each cluster was then selected and used to examine the ability to identify each respective tauopathy when comparing against all 127 cases together while controlling for age at death and gender. Using binary logistic regression analysis, CCL21 significantly correlated with a positive diagnosis of CTE (OR = 1.206, *p* < 0.001) independently of age at death (OR = 0.944, *p* = 0.036) or gender (OR = 0.00, *p* = 0.997). Next, it was observed that FLT3L, although not reaching significance, trended towards being able to predict AD (OR = 1.13, *p* = 0.054) independently of age at death (OR = 1.109, *p* < 0.001) or gender (OR = 2.679, *p* = 0.42). Cluster 3’s IL13 demonstrated significant ability to identify PSP (OR = 1.313, *p* < 0.001), independently of age at death (OR = 0.970, *p* = 0.276), or gender (OR = 2.076, *p* = 0.183). Unlike Clusters 1–3, the protein with the highest AUC in Cluster 4 was not the top protein in the PLS analysis. LIF had the highest Cluster 4 AUC, but IL1β was the top PLS correlated protein. However, neither IL1β (OR = 0.965, *p* = 0.816) or LIF (OR = 1.244, *p* = 0.232) correlated with a diagnosis of CBD. Similarly, for Cluster 5, GMCSF had the highest AUC but VEGFA was the top PLS correlated protein. While VEGFA (OR = 1.046, *p* = 0.246) was not correlated with a diagnosis of AGD, GMCSF was correlated with a diagnosis of AGD (OR = 5.148, *p* = 0.007) independently of age (OR = 1.132, *p* < 0.001) and gender (OR = 0.776, *p* = 0.676).

Binary logistic regression for the male only top associated proteins was then preformed as a sub-analysis. CCL21 was still significantly correlated with a diagnosis of CTE (OR = 1.206, *p* < 0.001), independently of age at death (OR = 0.944, *p* = 0.036). Additionally, IL13 again demonstrated significant ability to identify PSP (OR = 1.439, *p* = 0.001), independently of age at death (OR = 0.992, *p* = 0.850). Neither Cluster 2’s IL23 (OR = 1.007, *p* = 0.206), nor CCL2 (OR = 1.006, *p* = 0.456), demonstrate significant ability to identify AD. However, FLT3L was again able to predict AD in the male only cases (OR = 1.208, *p* = 0.039), independently of age at death (OR = 1.102, *p* = 0.019). Finally, CXCL9 was not significant for CBD (OR = 0.946, *p* = 0.220), or AGD (OR = 0.987, *p* = 0.620).

### CCL21 was able to identify CTE using postmortem CSF

**Fig. 4:**
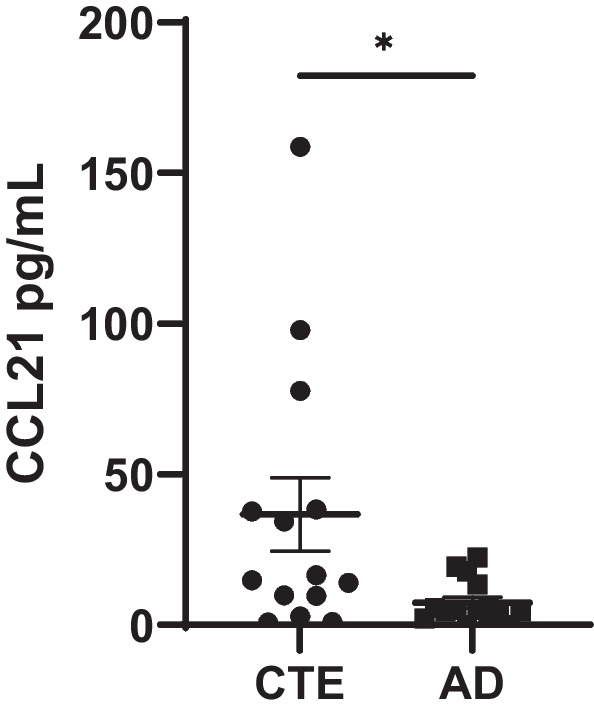
CCL21 is elevated in the CSF in CTE. To validate PLS results and determine if candidate proteins can be used as biomarkers as fluid biomarkers, the top distinct protein for CTE, CCL21, was measured in postmortem CSF from individuals with AD and CTE. CCL21 concentrations were higher in CTE compared to CTE as measured with a Mann–Whitney test (*p < 0.05). Each dot represents 1 case. Error bars represent mean ± SEM

Finally, to examine if the results taken from the brain homogenate can be extrapolated to fluids such as CSF, postmortem CSF from individuals with AD and CTE was obtained and a CCL21 ELISA was performed. When comparing the total concentrations, individuals with CTE were observed to have significantly more CCL21 compared to individuals with AD (*p* = 0.02) (Fig. 4). A sub-analysis using just the male cases also demonstrated that CCL21 trended towards elevation in CTE compared to AD (*p* = 0.056) (Additional file 1: Fig. S1).

## Discussion

Here we have shown that the complex neuroinflammatory response that occurs during disease might provide additional insight into unique disease mechanisms. Additionally, the neuroinflammatory response might be a useful source of candidate biomarkers to help identify tauopathies during life. Using a 71 immune-related protein multiplex ELISA panel, a PLS regression model was able to identify 5 distinct clusters of proteins that each correlated with a unique tauopathy. CTE, AD, and PSP had the highest overall correlation with their respective clusters. Although CBD and AGD had the strongest correlation with cluster 4 and 5 respectively, some overlap with other diseases was also observed. Using the top PLS hits, a ROC and binary logistic regression analysis demonstrated that CCL21 was the strongest predictor of CTE, FLT3L for AD, and IL13 for PSP. The strongest predictor proteins were consistent in both mixed gender and male only sub-analyses. Since there was less specificity in the clusters that belonged to CBD and AGD, a strong biomarker candidate was not observed. Finally, validation of the top PLS candidate proteins was performed in postmortem CSF, and it was observed that CCL21 was increased in CTE compared to AD. Overall, this work demonstrates that the neuroinflammatory signatures that are present among distinct tauopathies can be used to distinguish between diseases and could be an important source of novel biomarkers to aid in-life diagnosis.

Neurodegenerative pathologies are complex and might even change over the course of disease [27]. Therefore, it is likely a panel of biomarkers, consisting of serum or CSF sampling, PET or MRI imaging, and clinical symptoms will be needed to capture multiple diverse aspects of distinct neurodegenerative diseases. An important contribution of the current study is the identification of new candidate proteins that could be useful alongside of the other established biomarkers to help distinguish between neurodegenerative pathologies for a more specific in-life diagnosis. A panel consisting of CCL21, FLT3L, and IL-13 appeared to demonstrate significant power to distinguish between CTE, AD, and PSP, while CBD and AGD were a more ambiguous. To that end, it was demonstrated that CCL21 was also increased in the CSF in CTE compared to AD, suggesting the brain homogenate findings could translate to fluids and be viable clinical biomarkers. These results suggest that the inclusion of the candidate proteins into biomarker panels could aid as tools to identify and distinguish diseases in-life.

In addition to discovering possible novel biomarker candidates, by identifying specific sets of neuroinflammatory proteins linked to specific diseases, each clusters offers details on mechanistic processes that could be distinct among tauopathies. The added insight can help shape our understanding of pathogenesis and aid the design of disease specific therapies. Cluster 1 was found to correlate strongly for CTE and the top PLS hits were CCL21, CXCL5, CXCL13, GMCSF, and CCL17. Out of those top 5, CCL21 was the strongest driver of the cluster and was the strongest predictor of any of the proteins for their respective disease. CCL21, also known as 6CKine, is a chemokine that signals immune cell trafficking [28, 29]. The CCL21/CCR7 signaling axis has been shown to be an important component of T Cell extravasation into the brain [30]. CCL21 has also been shown to be a potent glial activator as well [31]. CCL21 has been associated with trauma-related injuries, suggesting the type of repetitive head trauma responsible inducing CTE could be driving a distinct CCL21 response [31, 32]. Interestingly, all the top 5 hits for CTE belong to the chemokine family of proteins suggesting some of the strongest CTE immune signals are related to recruiting immune cells. Previous findings have also observed the chemokine CCL11 (also called eotaxin) was elevated in CTE [19]. However, no increase in CCL11 was observed in the present study likely due to the advanced age of all the cases resulting in a ceiling effect.

Although AD is a highly studied disease, comparing the unique inflammatory response to other related tauopathies is less common. FLT3L, IL15, IL17E/IL25, IL17F, and CCL17 were the top proteins found to be distinctive for AD in Cluster 2. The most highly correlated protein to AD was FLT3L. FLT3L has several known effects relating to cell proliferation, metabolism, and leukocyte activation [33]. Several reports have detailed the importance of FLT3L in dendritic cell development [33] further highlighting a potential strong connection between AD and the immune system. Interestingly, FLT3L was also found to correlate with CSF tau concentration in cases of AD, Sjogren’s syndrome, and fibromyalgia [34]. The proinflammatory cytokine IL15, has also been previously suggested as a possible AD related biomarker, further validating the current findings [35].

After CCL21 in CTE, the correlation between IL13 and PSP was the next strongest. IL13 is an anti-inflammatory cytokine that is closely related to IL4 and shares several downstream pathways [36]. Typically, IL13 is involved in allergic inflammatory diseases or wound-healing events [36]. To our knowledge, there has not been any previous reports of IL13 involvement in PSP. Anti-inflammatory proteins have been found to be part of the neuropathologic process in other diseases, such as AD, but they were believed to be part of a protective feedback pathway [37]. Additional work will be needed to better understand how IL13 might be involved in PSP pathogenesis.

The final two diseases, CBD and AGD, demonstrated much less agreement with their respective clusters. ROC analysis for the top 5 PLS candidates for each disease did not reach significance, and only GMCSF correlated with AGD in a binary logistic regression. Although CBD and AGD had the highest correlation with Cluster 4 and 5, respectively, Fig. 1 demonstrated that there appeared to be moderate correlation with the other diseases as well. This suggests that the PLS candidate proteins identified for CBD and AGD are not as specific. It is possible that the anterior cingulate cortex was less affected in these diseases resulting in a more muted neurodegenerative response. Additionally, it is possible that CBD and AGD share more neuroimmune protein signatures to the other diseases as well. The present analysis does not compare absolute protein levels to a non-affected control case. Therefore, these results do not suggest that neurodegenerative proteins are not elevated in disease. Rather, they demonstrated that there was a lack of neuroimmune related proteins that could specifically identify AGD or CBD compared to CTE, AD, or PSP. Future work will be needed to more thoroughly investigate what other proteins could act as biomarkers for AGD or CBD to better distinguish them.

Although gender was controlled for during the PLS and binary logistic regressions, it is important to point out that the CTE group was composed entirely of males which could skew results. This was due to the current lack of females diagnosed with CTE, as many of the donated samples were derived from individuals who played American football, a sport dominated by males. To account for this skew, a sub-analysis with just males was performed. The results for CTE compared to the other tauopathies with just males was consistent with CCL21 as the most predictive CTE protein. This was also observed in the CSF sub-analysis. Additionally, IL13 was again the strongest predictive protein for PSP. However, when using just males, CCL2 and IL23 moved ahead of FLT3L for AD. When investigating the ability of the new top 2 proteins to predict AD, they were not significant, while consistent with the mixed gender analysis, FLT3L was predictive. This suggested that while CCL2 and IL23 might have some additional role in AD males, it is likely that removing half of the samples severely limited the power of the analysis and increased noise was added to the top associated proteins. Like the mixed gender full analysis, the male only sub-analysis did not find any predictive proteins for AGD or CBD. Therefore, since the most predictive protein for CTE (CCL21), AD (FLT3L), and PSP (IL13) was consistent across mixed and single gender analyses, these results provide support that the findings were not significantly affected by the CTE male only skew. Gender differences during neurodegeneration is an important topic and likely contributed to the results in some degree. However, a more comprehensive future study will be needed to tease apart the gender differences in a larger sample set.

While this current study is one of the most comprehensive to date looking at dozens of different proteins across 5 different diseases, there are likely many more differences not captured with the multiplex ELISA. More unbiased proteomic techniques like mass spectrometry would be useful to identify the full spectrum of differences present between diseases and identify additional targets. Absolute levels of each protein will also need to be compared to control cases to have a better understanding of changes during normal aging. Additionally, future studies will be needed to determine the cellular source of each protein to increase understanding of possible disease mechanisms and further validate the current findings. Finally, the current study only examined one brain region, the anterior cingulate cortex. This region was selected as it was affected in all diseases and offered the best chance to identify ptau related changes. However, the progression of ptau is distinct in each tauopathy. Therefore, future studies will be needed to examine the neuroimmune changes that occur in a region-by-region progression.

## Conclusion

In conclusion, here we have shown that there are unique neuroinflammatory pathways present among tauopathies that can provide greater insight into distinct mechanisms of disease. Additionally, these results also suggest that neuroinflammatory proteins are a good source of possible novel biomarker candidates to distinguish between tauopathies. CCL21, FLT3L, and IL13 are novel candidates that could be useful in future work to help better understand and differentiate CTE, AD, and PSP. Although it is unclear how the absolute levels of the proposed candidates differ from non-disease control cases, the current study is significant as it suggests novel markers that can help differentiate between related diseases and add increased disease specificity to other biomarker panels. These candidate proteins will likely be needed to be used in conjunction with a panel of already established proteins (including Aβ and ptau), imaging studies such as PET and MRI, and clinical measures to truly identify and capture the complexity of each disease in life.

## Supplementary Information

**Additional file 1**: **Figure S1. **CCL21 is still elevated in the CTE CSF in the male subset of cases. To determine if gender was driving the CSF related CCL21 elevation, only males from the CTE and AD groups were compared. Although the AD male sample size was reduced to 5 cases, CCL21 concentrations still trended towards significant increases in CTE as measured with a Mann–Whitney test. Each dot represents 1 case. Error bars represent mean ± SEM.

**Additional file 2**: **Table S1**. Full Association List of All Cases.

**Additional file 3**: **Table S2**. Full Association List of Males Only.

## Data Availability

The datasets used and analyzed during the current study are available from the corresponding author on reasonable request.

## Abbreviations

AD: Alzheimer’s disease
AGD: Argyrophilic grain disease
AUC: Area under the curve
CBD: Corticobasal degeneration
CERAD: Consortium to Establish a Registry for Alzheimer’s Disease
CSF: Cerebrospinal fluid
CTE: Chronic traumatic encephalopathy
PLS: Partial least square
PSP: Progressive supranuclear palsy
ptau: Hyperphosphorylated tau
ROC: Receiver operating characteristic
SVD: Singular value decomposition

## References

[1] Arendt T, Stieler JT, Holzer M (2016). Tau and tauopathies. Brain Res Bull.

[2] McKeith IG (2006). Consensus guidelines for the clinical and pathologic diagnosis of dementia with Lewy bodies (DLB): report of the Consortium on DLB International Workshop. J Alzheimers Dis.

[3] Love S, Louis D, Ellison DW (2008). Greenfield’s neuropathology, 2-volume set.

[4] Mackenzie IR, Neumann M, Bigio EH, Cairns NJ, Alafuzoff I, Kril J, Kovacs GG, Ghetti B, Halliday G, Holm IE (2010). Nomenclature and nosology for neuropathologic subtypes of frontotemporal lobar degeneration: an update. Acta Neuropathol.

[5] Montine TJ, Phelps CH, Beach TG, Bigio EH, Cairns NJ, Dickson DW, Duyckaerts C, Frosch MP, Masliah E, Mirra SS (2012). National Institute on Aging-Alzheimer's Association guidelines for the neuropathologic assessment of Alzheimer's disease: a practical approach. Acta Neuropathol.

[6] McKee AC, Cairns NJ, Dickson DW, Folkerth RD, Dirk Keene C, Litvan I, Perl DP, Stein TD, Vonsattel J-P, Stewart W (2015). The first NINDS/NIBIB consensus meeting to define neuropathological criteria for the diagnosis of chronic traumatic encephalopathy. Acta Neuropathol.

[7] Bieniek KF, Cairns NJ, Crary JF, Dickson DW, Folkerth RD, Keene CD, Litvan I, Perl DP, Stein TD, Vonsattel JP (2021). The second NINDS/NIBIB consensus meeting to define neuropathological criteria for the diagnosis of chronic traumatic encephalopathy. J Neuropathol Exp Neurol.

[8] Roemer SF, Grinberg LT, Crary JF, Seeley WW, McKee AC, Kovacs GG, Beach TG, Duyckaerts C, Ferrer IA, Gelpi E (2022). Rainwater Charitable Foundation criteria for the neuropathologic diagnosis of progressive supranuclear palsy. Acta Neuropathol.

[9] Butler M, Dixon E, Stein TD, Alvarez VE, Huber B, Buckland ME, McKee AC, Cherry JD (2022). Tau pathology in chronic traumatic encephalopathy is primarily neuronal. J Neuropathol Exp Neurol.

[10] Alosco ML, Tripodis Y, Fritts NG, Heslegrave A, Baugh CM, Conneely S, Mariani M, Martin BM, Frank S, Mez J (2018). Cerebrospinal fluid tau, Abeta, and sTREM2 in Former National Football League Players: modeling the relationship between repetitive head impacts, microglial activation, and neurodegeneration. Alzheimers Dement.

[11] Turk KW, Geada A, Alvarez VE, Xia W, Cherry JD, Nicks R, Meng G, Daley S, Tripodis Y, Huber BR (2022). A comparison between tau and amyloid-beta cerebrospinal fluid biomarkers in chronic traumatic encephalopathy and Alzheimer disease. Alzheimers Res Ther.

[12] Tissot C, Therriault J, Kunach P, Benedet AL, Pascoal TA, Ashton NJ, Karikari TK, Servaes S, Lussier FZ, Chamoun M (2022). Comparing tau status determined via plasma pTau181, pTau231 and [(18)F]MK6240 tau-PET. EBioMedicine.

[13] Paolicelli R, Sierra A, Stevens B, Temblay ME, Aguzzi A, Ajami B, Amit I, Audinat E, Bechmann I, Bennett M, et al. Defining microglial states and nomenclature: a roadmap to 2030. Cell. 2022, Preprint. Available at SSRN: https://ssrn.com/abstract=4065080.

[14] Escartin C, Galea E, Lakatos A, O'Callaghan JP, Petzold GC, Serrano-Pozo A, Steinhauser C, Volterra A, Carmignoto G, Agarwal A (2021). Reactive astrocyte nomenclature, definitions, and future directions. Nat Neurosci.

[15] Olah M, Menon V, Habib N, Taga MF, Ma Y, Yung CJ, Cimpean M, Khairallah A, Coronas-Samano G, Sankowski R (2020). Single cell RNA sequencing of human microglia uncovers a subset associated with Alzheimer's disease. Nat Commun.

[16] Chancellor KB, Chancellor SE, Duke-Cohan JE, Huber BR, Stein TD, Alvarez VE, Okaty BW, Dymecki SM, McKee AC (2021). Altered oligodendroglia and astroglia in chronic traumatic encephalopathy. Acta Neuropathol.

[17] Sharma A, Song W-M, Farrell K, Whitney K, Zhang B, Crary JF, Pereira AC. Single-cell atlas of progressive supranuclear palsy reveals a distinct hybrid glial cell population. bioRxiv 2021.

[18] Sadick JS, O'Dea MR, Hasel P, Dykstra T, Faustin A, Liddelow SA (2022). Astrocytes and oligodendrocytes undergo subtype-specific transcriptional changes in Alzheimer's disease. Neuron.

[19] Cherry JD, Stein TD, Tripodis Y, Alvarez VE, Huber BR, Au R, Kiernan PT, Daneshvar DH, Mez J, Solomon TM (2017). CCL11 is increased in the CNS in chronic traumatic encephalopathy but not in Alzheimer's disease. PLoS ONE.

[20] Villeda SA, Luo J, Mosher KI, Zou B, Britschgi M, Bieri G, Stan TM, Fainberg N, Ding Z, Eggel A (2011). The ageing systemic milieu negatively regulates neurogenesis and cognitive function. Nature.

[21] Cherry JD, Meng G, Daley S, Xia W, Svirsky S, Alvarez VE, Nicks R, Pothast M, Kelley H, Huber B (2020). CCL2 is associated with microglia and macrophage recruitment in chronic traumatic encephalopathy. J Neuroinflam.

[22] McKee AC, Stern RA, Nowinski CJ, Stein TD, Alvarez VE, Daneshvar DH, Lee HS, Wojtowicz SM, Hall G, Baugh CM (2013). The spectrum of disease in chronic traumatic encephalopathy. Brain.

[23] Tekin S, Mega MS, Masterman DM, Chow T, Garakian J, Vinters HV, Cummings JL (2001). Orbitofrontal and anterior cingulate cortex neurofibrillary tangle burden is associated with agitation in Alzheimer disease. Ann Neurol.

[24] Kovacs GG, Budka H (2010). Current concepts of neuropathological diagnostics in practice: neurodegenerative diseases. Clin Neuropathol.

[25] Robinson JL, Yan N, Caswell C, Xie SX, Suh E, Van Deerlin VM, Gibbons G, Irwin DJ, Grossman M, Lee EB (2020). Primary tau pathology, not copathology, correlates with clinical symptoms in PSP and CBD. J Neuropathol Exp Neurol.

[26] Ferrer I, Santpere G, van Leeuwen FW (2008). Argyrophilic grain disease. Brain.

[27] Cherry JD, Kim SH, Stein TD, Pothast MJ, Nicks R, Meng G, Huber BR, Mez J, Alosco ML, Tripodis Y (2020). Evolution of neuronal and glial tau isoforms in chronic traumatic encephalopathy. Brain Pathol.

[28] Yoshida R, Imai T, Hieshima K, Kusuda J, Baba M, Kitaura M, Nishimura M, Kakizaki M, Nomiyama H, Yoshie O (1997). Molecular cloning of a novel human CC chemokine EBI1-ligand chemokine that is a specific functional ligand for EBI1, CCR7. J Biol Chem.

[29] Forster R, Schubel A, Breitfeld D, Kremmer E, Renner-Muller I, Wolf E, Lipp M (1999). CCR7 coordinates the primary immune response by establishing functional microenvironments in secondary lymphoid organs. Cell.

[30] Wang W, Liu E, Li X, Chen S, Pang S, Zhang Y (2022). CCL21 contributes to Th17 cell migration in neuroinflammation in obese mice following lead exposure. Toxicol Lett.

[31] Li Y, Cao T, Ritzel RM, He J, Faden AI, Wu J (2020). Dementia, depression, and associated brain inflammatory mechanisms after spinal cord injury. Cells.

[32] Chen Y, Liang L, Cao S, Hou G, Zhang Q, Ma H, Shi B (2020). Serum CCL21 as a potential biomarker for cognitive impairment in spinal cord injury. Biomed Res Int.

[33] Wilson KR, Villadangos JA, Mintern JD (2021). Dendritic cell Flt3—regulation, roles and repercussions for immunotherapy. Immunol Cell Biol.

[34] Dehlin M, Bjersing J, Erlandsson M, Andreasen N, Zetterberg H, Mannerkorpi K, Bokarewa M (2013). Cerebrospinal Flt3 ligand correlates to tau protein levels in primary Sjogren's syndrome. Scand J Rheumatol.

[35] Bishnoi RJ, Palmer RF, Royall DR (2015). Serum interleukin (IL)-15 as a biomarker of Alzheimer's disease. PLoS ONE.

[36] Liang HE, Reinhardt RL, Bando JK, Sullivan BM, Ho IC, Locksley RM (2012). Divergent expression patterns of IL-4 and IL-13 define unique functions in allergic immunity. Nat Immunol.

[37] Cherry JD, Olschowka JA, O’Banion MK (2015). Arginase 1+ microglia reduce Aβ plaque deposition during IL-1β-dependent neuroinflammation. J Neuroinflam.

