# Supplementary figures and images for: Neuroimmune proteins can differentiate between tauopathies
Source: J Neuroinflammation. 2022 Nov 19;19:278. doi: 10.1186/s12974-022-02640-6 (PMC9675129; doi:10.1186/s12974-022-02640-6)

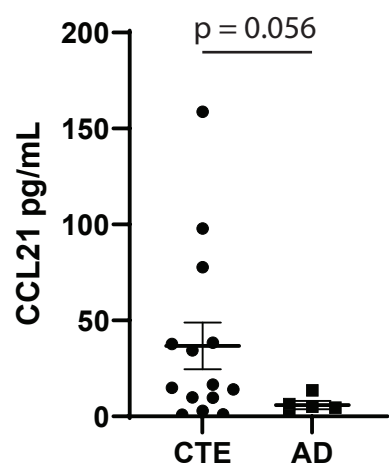

Supplement: Supplementary file 1 — Additional file 1: Figure S1. CCL21 is still elevated in the CTE CSF in the male subset of cases. To determine if gender was driving the CSF related CCL21 elevation, only males from the CTE and AD groups were compared. Although the AD male sample size was reduced to 5 cases, CCL21 concentrations still trended towards significant increases in CTE as measured with a Mann–Whitney test. Each dot represents 1 case. Error bars represent mean ± SEM. [file 12974_2022_2640_MOESM1_ESM.pdf]
