# Supplementary material for: Neuroimmune proteins can differentiate between tauopathies
Source: J Neuroinflammation. 2022 Nov 19;19:278. doi: 10.1186/s12974-022-02640-6 (PMC9675129; doi:10.1186/s12974-022-02640-6)
Supplement: Supplementary file 2 — Additional file 2: Table S1. Full Association List of All Cases. [file 12974_2022_2640_MOESM2_ESM.docx]

| **Table S1 Full Association List of All Cases** | | | | |
| --- | --- | --- | --- | --- |
| **Cluster 1** | **Cluster 2** | **Cluster 3** | **Cluster 4** | **Cluster 5** |
| CCL21 (0.36) | FLT3L (0.31) | IL13 (0.37) | IL1β (0.37) | VEGFA (0.33) |
| CXCL5 (0.27) | IL17F (0.25) | SCF (0.25) | CCL22 (0.37) | CCL2 (0.32) |
| CXCL13 (0.24) | CCL17 (0.23) | CXCL13 (0.25) | CXCL9 (0.21) | CXCL9 (0.31) |
| GMCSF (0.24) | IL15 (0.23) | CXCL9 (0.24) | LIF (0.2) | FLT3L (0.28) |
| CCL17 (0.23) | IL17E/IL25 (0.21) | IL3 (0.24) | VEGFA (0.19) | GMCSF (0.23) |
| CX3CL1 (0.21) | CXCL5 (0.21) | IL12p70 (0.24) | CXCL5 (0.19) | TRIAL (0.22) |
| TPO (0.21) | CCL8 (0.21) | IL17E/IL25 (0.22) | CCL11 (0.18) | TSLP (0.19) |
| CXCL10 (0.18) | IL3 (0.19) | IL17F (0.2) | IL10 (0.18) | PDGFAA (0.18) |
| IL8 (0.18) | SCF (0.19) | CCL13 (0.19) | CCL27 (0.18) | CCL5 (0.17) |
| CCL2 (0.17) | IL16 (0.16) | TRIAL (0.17) | CCL21 (0.18) | IL10 (0.16) |
| IL10 (0.17) | PDGFAA (0.16) | IL23 (0.17) | IL4 (0.17) | CX3CL1 (0.16) |
| CCL27 (0.17) | CCL22 (0.16) | IL28A (0.16) | CXCL12 (0.16) | PDGFAB/BB (0.16) |
| IL27 (0.16) | IL33 (0.16) | CCL27 (0.16) | IL9 (0.16) | IL1α (0.16) |
| PDGFAA (0.16) | CCL7 (0.16) | TGFα (0.15) | IL15 (0.16) | IFNα2 (0.15) |
| IL12p70 (0.16) | IL21 (0.16) | CXCL5 (0.14) | IL17E/IL25 (0.15) | IL15 (0.14) |
| IL33 (0.16) | GROα (0.16) | LIF (0.13) | CCL1 (0.14) | FGF2 (0.14) |
| IL12p40 (0.15) | IL23 (0.16) | TNFβ (0.13) | TGFα (0.14) | IL9 (0.13) |
| TNFα (0.13) | TPO (0.15) | IL27 (0.13) | MIP1e (0.13) | CSF1 (0.13) |
| IL17E/IL25 (0.13) | CCL1 (0.14) | IL12p40 (0.12) | IL1Rα (0.13) | IL1β (0.13) |
| IL23 (0.13) | CCL27 (0.14) | CCL11 (0.12) | TPO (0.13) | IL8 (0.13) |
| IL3 (0.13) | CSF1 (0.14) | GMCSF (0.12) | TRIAL (0.13) | IL4 (0.12) |
| IL16 (0.13) | CXCL10 (0.14) | FLT3L (0.12) | IL8 (0.12) | CCL22 (0.12) |
| IL28A (0.12) | IL18 (0.13) | CSF1 (0.11) | sCD40L (0.12) | IL6 (0.11) |
| CCL13 (0.12) | TSLP (0.13) | IL9 (0.11) | CCL3 (0.12) | IL17E/IL25 (0.11) |
| IL18 (0.11) | IL6 (0.12) | CCL21 (0.11) | SCF (0.11) | IL3 (0.11) |
| IL1β (0.1) | IL4 (0.12) | IFNy (0.1) | IL1α (0.11) | CCL13 (0.1) |
| CSF1 (0.1) | CCL2 (0.12) | CCL7 (0.1) | CCL17 (0.1) | IL28A (0.1) |
| IL1α (0.09) | IL27 (0.12) | IFNα2 (0.1) | CCL5 (0.1) | IFNy (0.1) |
| GROα (0.09) | TGFα (0.1) | CCL5 (0.09) | PDGFAA (0.09) | sCD40L (0.1) |
| IL6 (0.09) | TRIAL (0.1) | CXCL12 (0.09) | IL12p40 (0.09) | TNFα (0.1) |
| IFNα2 (0.08) | TNFβ (0.1) | IL1α (0.09) | CCL2 (0.09) | IL16 (0.1) |
| LIF (0.08) | IL8 (0.1) | IL16 (0.09) | TNFβ (0.09) | CXCL12 (0.09) |
| IL15 (0.08) | IL9 (0.1) | IL4 (0.09) | TNFα (0.09) | CCL3 (0.09) |
| TRIAL (0.08) | EGF (0.1) | CCL3 (0.08) | IL23 (0.09) | TGFα (0.07) |
| IL13 (0.08) | GCSF (0.09) | CCL17 (0.08) | CCL13 (0.08) | IL12p70 (0.07) |
| IL21 (0.08) | CCL3 (0.09) | IL6 (0.08) | IL28A (0.08) | MIP1e (0.07) |
| TNFβ (0.07) | CCL11 (0.08) | PDGFAA (0.07) | FLT3L (0.07) | CCL17 (0.06) |
| CXCL9 (0.07) | CXCL12 (0.08) | EGF (0.06) | PDGFAB/BB (0.07) | IL27 (0.06) |
| IL1Rα (0.07) | IL1α (0.08) | CXCL10 (0.06) | IL6 (0.07) | CXCL5 (0.06) |
| CCL24 (0.07) | CXCL13 (0.08) | IL33 (0.06) | CXCL10 (0.07) | SCF (0.05) |
| CCL1 (0.06) | IL28A (0.07) | VEGFA (0.06) | TSLP (0.07) | CCL7 (0.05) |
| sCD40L (0.06) | IL10 (0.07) | IL1β (0.06) | IL17F (0.07) | CCL27 (0.05) |
| EGF (0.06) | CXCL9 (0.07) | TPO (0.06) | CCL7 (0.06) | IL18 (0.05) |
| GCSF (0.06) | sCD40L (0.07) | CCL2 (0.05) | FGF2 (0.06) | CCL8 (0.05) |
| IL17F (0.05) | CCL21 (0.06) | CCL8 (0.05) | GROα (0.05) | TPO (0.04) |
| FLT3L (0.05) | IL12p70 (0.06) | IL18 (0.05) | CCL24 (0.05) | LIF (0.04) |
| FGF2 (0.05) | LIF (0.05) | GCSF (0.05) | IL33 (0.05) | IL17F (0.03) |
| SCF (0.05) | IL1Rα (0.05) | GROα (0.04) | GMCSF (0.05) | CCL11 (0.03) |
| CCL5 (0.04) | MIP1e (0.04) | MIP1e (0.04) | IFNα2 (0.04) | CCL21 (0.03) |
| IL4 (0.04) | FGF2 (0.04) | IL8 (0.04) | IL21 (0.04) | CXCL13 (0.03) |
| MIP1e (0.04) | IL13 (0.03) | IL15 (0.04) | IL27 (0.04) | CCL24 (0.02) |
| IFNy (0.03) | IFNα2 (0.03) | IL21 (0.03) | IL18 (0.03) | TNFβ (0.02) |
| CCL22 (0.03) | VEGFA (0.03) | IL1Rα (0.03) | IL3 (0.03) | GCSF (0.02) |
| CCL3 (0.03) | IL12p40 (0.03) | CCL1 (0.03) | IL12p70 (0.03) | CCL1 (0.02) |
| IL9 (0.02) | IFNy (0.02) | PDGFAB/BB (0.03) | IL13 (0.02) | GROα (0.01) |
| CCL11 (0.02) | PDGFAB/BB (0.02) | CCL24 (0.03) | IFNy (0.02) | IL23 (0.01) |
| PDGFAB/BB (0.02) | CCL5 (0.02) | sCD40L (0.02) | CCL8 (0.02) | IL1Rα (0.01) |
| CXCL12 (0.01) | TNFα (0.01) | CX3CL1 (0.02) | GCSF (0.01) | CXCL10 (0.01) |
| CCL7 (0.01) | CCL13 (0.01) | FGF2 (0.02) | EGF (0.01) | IL13 (0) |
| CCL8 (0.01) | CX3CL1 (0.01) | CCL22 (0.02) | CSF1 (0.01) | EGF (0) |
| VEGFA (0.01) | GMCSF (0) | IL10 (0.01) | IL16 (0.01) | IL33 (0) |
| TGFα (0) | IL1β (0) | TSLP (0.01) | CXCL13 (0.01) | IL12p40 (0) |
| TSLP (0) | CCL24 (0) | TNFα (0) | CX3CL1 (0) | IL21 (0) |

Values presented as: protein name (correlation statistic)
