# Supplementary material for: Neuroimmune proteins can differentiate between tauopathies
Source: J Neuroinflammation. 2022 Nov 19;19:278. doi: 10.1186/s12974-022-02640-6 (PMC9675129; doi:10.1186/s12974-022-02640-6)
Supplement: Supplementary file 3 — Additional file 3: Table S2. Full Association List of Males Only. [file 12974_2022_2640_MOESM3_ESM.docx]

| **Table S2 Full Association List of Males Only** | | | | |
| --- | --- | --- | --- | --- |
| **Cluster 1** | **Cluster 2** | **Cluster 3** | **Cluster 4** | **Cluster 5** |
| CCL21 (0.31) | IL23 (0.27) | IL13 (0.3) | CXCL9 (0.35) | CXCL9 (0.31) |
| CXCL5 (0.25) | CCL2 (0.24) | CXCL13 (0.27) | CCL22 (0.26) | IL17F (0.3) |
| GMCSF (0.25) | FLT3L (0.24) | IL17E/IL25 (0.23) | TRIAL (0.24) | TRIAL (0.27) |
| CCL17 (0.23) | CXCL5 (0.23) | SCF (0.22) | IL1β (0.22) | IL1α (0.25) |
| CXCL13 (0.23) | IL17E/IL25 (0.22) | IL12p70 (0.22) | CXCL5 (0.22) | CCL5 (0.23) |
| CX3CL1 (0.22) | CCL17 (0.21) | IL17F (0.21) | IL10 (0.21) | IL18 (0.23) |
| TPO (0.2) | CCL27 (0.21) | TGFα (0.21) | TPO (0.2) | IL1Rα (0.23) |
| IL10 (0.2) | IL15 (0.2) | IL28A (0.2) | IL17E/IL25 (0.19) | SCF (0.22) |
| CXCL10 (0.19) | IL17F (0.2) | IL12p40 (0.2) | IL1α (0.18) | GMCSF (0.22) |
| IL27 (0.18) | GROα (0.19) | IL3 (0.18) | IL15 (0.18) | CCL22 (0.19) |
| IL12p40 (0.18) | IL8 (0.19) | CCL27 (0.18) | VEGFA (0.18) | FLT3L (0.18) |
| IL12p70 (0.18) | TPO (0.18) | TRIAL (0.17) | IL4 (0.17) | TSLP (0.17) |
| CCL27 (0.16) | CCL8 (0.17) | FLT3L (0.17) | CCL27 (0.15) | CCL24 (0.16) |
| IL33 (0.16) | IL33 (0.17) | CXCL9 (0.17) | CCL1 (0.14) | IL33 (0.16) |
| IL3 (0.16) | SCF (0.16) | IL15 (0.17) | CCL13 (0.14) | CXCL5 (0.15) |
| IL8 (0.16) | PDGFAA (0.16) | CSF1 (0.17) | MIP1e (0.14) | CCL17 (0.14) |
| PDGFAA (0.15) | CCL22 (0.15) | PDGFAA (0.15) | IL13 (0.14) | CCL2 (0.13) |
| CCL2 (0.15) | IL21 (0.15) | LIF (0.14) | LIF (0.13) | sCD40L (0.12) |
| TNFα (0.14) | IL6 (0.15) | CCL21 (0.13) | IL28A (0.13) | IL3 (0.12) |
| IL1β (0.13) | IL16 (0.13) | IL9 (0.13) | PDGFAA (0.13) | CCL21 (0.11) |
| IL17E/IL25 (0.12) | IL4 (0.13) | CCL3 (0.13) | FLT3L (0.13) | CSF1 (0.11) |
| IL28A (0.12) | IL27 (0.12) | CCL13 (0.13) | IL16 (0.13) | VEGFA (0.11) |
| IL18 (0.12) | IL18 (0.12) | FGF2 (0.12) | CXCL12 (0.13) | IL15 (0.11) |
| IFNα2 (0.11) | PDGFAB/BB (0.12) | IL16 (0.12) | CCL2 (0.12) | IL6 (0.11) |
| CCL13 (0.11) | CCL7 (0.11) | CCL11 (0.11) | PDGFAB/BB (0.12) | PDGFAA (0.1) |
| IL13 (0.11) | CXCL13 (0.11) | CXCL5 (0.11) | TGFα (0.11) | CCL27 (0.09) |
| IL23 (0.11) | CCL24 (0.11) | CCL22 (0.11) | IL18 (0.11) | IL4 (0.09) |
| TNFβ (0.11) | CCL1 (0.11) | IL27 (0.11) | IFNy (0.11) | CCL11 (0.09) |
| IL16 (0.1) | CCL3 (0.11) | CX3CL1 (0.11) | CCL11 (0.11) | IFNα2 (0.09) |
| TRIAL (0.1) | CXCL10 (0.1) | GMCSF (0.1) | CCL24 (0.11) | CXCL12 (0.09) |
| IL1Rα (0.1) | EGF (0.1) | CCL24 (0.1) | EGF (0.11) | PDGFAB/BB (0.09) |
| IL1α (0.1) | TRIAL (0.1) | IL18 (0.09) | CCL5 (0.1) | IL12p40 (0.09) |
| CXCL9 (0.09) | TNFβ (0.1) | CCL17 (0.09) | SCF (0.1) | LIF (0.08) |
| GROα (0.09) | CCL5 (0.1) | IFNα2 (0.09) | IL21 (0.1) | IL13 (0.08) |
| EGF (0.09) | GCSF (0.1) | TNFβ (0.08) | CXCL10 (0.09) | IL8 (0.07) |
| IL15 (0.08) | sCD40L (0.09) | CCL2 (0.08) | IL1Rα (0.09) | TGFα (0.07) |
| CSF1 (0.08) | IL9 (0.09) | CXCL10 (0.08) | GMCSF (0.09) | TNFβ (0.07) |
| IL21 (0.08) | CXCL9 (0.08) | IFNy (0.08) | FGF2 (0.09) | EGF (0.06) |
| LIF (0.08) | CXCL12 (0.07) | IL23 (0.08) | IL33 (0.09) | IL23 (0.06) |
| sCD40L (0.07) | IL3 (0.07) | CCL1 (0.07) | CSF1 (0.08) | IL28A (0.06) |
| IL6 (0.07) | IL12p70 (0.07) | CXCL12 (0.07) | TNFα (0.08) | IL21 (0.05) |
| CCL11 (0.07) | CSF1 (0.06) | GCSF (0.06) | sCD40L (0.07) | IL17E/IL25 (0.05) |
| GCSF (0.06) | CX3CL1 (0.06) | IL6 (0.06) | IL27 (0.07) | CX3CL1 (0.04) |
| FLT3L (0.06) | GMCSF (0.05) | IL33 (0.06) | CCL7 (0.07) | FGF2 (0.04) |
| CCL24 (0.05) | TNFα (0.05) | CCL7 (0.05) | IL3 (0.06) | CCL8 (0.04) |
| CCL1 (0.05) | FGF2 (0.05) | IL1α (0.05) | IL8 (0.05) | IL9 (0.04) |
| CCL22 (0.05) | CCL21 (0.05) | CCL8 (0.05) | TSLP (0.05) | GCSF (0.04) |
| IL4 (0.05) | TSLP (0.04) | IL1β (0.05) | TNFβ (0.04) | CCL1 (0.03) |
| IL9 (0.05) | LIF (0.04) | VEGFA (0.04) | IL12p70 (0.04) | IL27 (0.03) |
| IFNy (0.04) | IFNα2 (0.04) | IL8 (0.03) | CCL3 (0.04) | CXCL13 (0.03) |
| MIP1e (0.04) | CCL13 (0.03) | GROα (0.03) | IL17F (0.04) | CCL13 (0.02) |
| IL17F (0.04) | IL10 (0.03) | TPO (0.03) | CCL21 (0.04) | IL10 (0.02) |
| FGF2 (0.03) | CCL11 (0.03) | TSLP (0.03) | IL23 (0.03) | GROα (0.02) |
| CCL5 (0.03) | IFNy (0.03) | IL4 (0.02) | GROα (0.03) | TPO (0.02) |
| CCL7 (0.02) | TGFα (0.03) | IL21 (0.02) | IL9 (0.03) | IFNy (0.01) |
| SCF (0.02) | IL28A (0.03) | CCL5 (0.02) | CCL17 (0.02) | CCL7 (0.01) |
| PDGFAB/BB (0.02) | VEGFA (0.03) | IL10 (0.02) | IL6 (0.01) | TNFα (0.01) |
| CCL8 (0.01) | IL1Rα (0.02) | PDGFAB/BB (0.01) | CCL8 (0.01) | IL1β (0.01) |
| CCL3 (0.01) | MIP1e (0.01) | TNFα (0.01) | IFNα2 (0.01) | IL12p70 (0.01) |
| TSLP (0.01) | IL1β (0.01) | EGF (0.01) | GCSF (0.01) | CCL3 (0.01) |
| CXCL12 (0.01) | IL1α (0.01) | IL1Rα (0.01) | CXCL13 (0.01) | MIP1e (0.01) |
| VEGFA (0) | IL13 (0.01) | MIP1e (0) | CX3CL1 (0.01) | CXCL10 (0.01) |
| TGFα (0) | IL12p40 (0.01) | sCD40L (0) | IL12p40 (0) | IL16 (0) |

Values presented as: protein name (correlation statistic)
